# First report on HIV molecular epidemiology in a native community from Argentina reveals transmission clades in the context of a unique HLA background

**DOI:** 10.1186/1742-4690-9-S2-P155

**Published:** 2012-09-13

**Authors:** D Monaco, D Dilernia, L Yue, M Quipildor, A Di Paolo, E Hunter, H Salomon

**Affiliations:** 1 Argentinean Reference Center for AIDS, Buenos Aires, Argentina; 2 Emory Vaccine Center, Atlanta, GA, USA; 3 Hospital San Vicente de Paul, San Ramón de la Nueva Oran, Argentina

## Background

The Department of Orán is located on the North of Argentina and is populated by native settlements and urbanized areas. During the last twelve years, an increasing number of HIV infections have been detected. We previously reported a highly limited HLA diversity with a reduced number of HLA-A and B alleles present at high prevalence. The objective of the present study was to characterize the HIV epidemic through full-length genome sequencing of the virus in this population.

## Methods

Samples from 147 HIV positive individuals collected in three campaigns to Orán were analyzed. Viral load, CD4-count and drug resistance were assessed and reported to the clinicians. Limiting-dilution full genome sequencing was performed from plasma on 65 of the samples. Viral diversity was analyzed by recombination (SimPlot) and Neighbor-Joining trees with bootstrap. HLA-A, B and C were characterized by SBT.

## Results

HLA typing showed a limited genetic diversity even at the four-digits high-resolution typing. This allele distribution resembles the one reported previously for this community with two-digits and confirms that the individuals included were in fact native. SimPlot analysis of the 65 complete and nearly-complete genomes showed a high prevalence of BF recombinants(70.77%) and the presence of subtype B(23.08%), C(3.08%) and F(3.08%) virus. 7 main recombination structures were found repeatedly in not-epidemiologically-linked individuals within highly supported monophyletic clades suggesting transmission of these particular viral strains within the community.

## Conclusion

This HIV epidemic seems to be characterized by a few introductions of viral variants that had spread on the community. Most of them have been BF recombinants with particular recombination structures. This work will allow us to further investigate the impact of the genetic features of this native community on viral evolution but also has facilitated the access of these individuals to clinical studies and counseling.

